# Interocular symmetry in dynamic retinal vessel analysis among healthy adults

**DOI:** 10.17305/bb.2026.14216

**Published:** 2026-05-08

**Authors:** Michael Mendes Wefelnberg, Freerk T Baumann, Henning Guthoff, Damir Zubac

**Affiliations:** 1 Department I of Internal Medicine, Center for Integrated Oncology Aachen Bonn Cologne Düsseldorf (CIO ABCD), Faculty of Medicine and University Hospital Cologne, University of Cologne, Cologne, Germany; 2 Clinic III for Internal Medicine, Faculty of Medicine and University Hospital Cologne, University of Cologne, Cologne, Germany; 3 Center for Cardiovascular Medicine Aachen Bonn Cologne Düsseldorf (CCM ABCD), Aachen, Bonn, Cologne and Düsseldorf, Germany

**Keywords:** Microvascular circulation, oxygen kinetics, flow mediated dilation

## Abstract

Dynamic retinal vessel analysis is a non-invasive approach for assessing retinal microvascular endothelial function, yet the extent to which eye selection, interocular variability, and systemic physiological factors influence dynamic retinal vessel analyzer (DRVA)-derived biomarkers remains insufficiently defined. This prospective methodological study aimed to evaluate the interocular symmetry and absolute and relative reliability of arterial flicker-induced dilation (aFID), venular flicker-induced dilation (vFID), and arteriolar constriction (aCON), and to determine whether these parameters are moderated by eye dominance, peak oxygen uptake (˙̇O_2_ peak), or intraocular pressure (IOP) in healthy individuals. Twenty apparently healthy adults completed two laboratory visits. During the first visit, aerobic capacity was assessed by cardiopulmonary exercise testing until volitional exhaustion. During the second visit, IOP, resting blood pressure, eye dominance, and retinal vascular endothelial function were assessed using DRVA in both eyes in randomized order. Interocular differences were examined using paired comparisons, Bland–Altman analysis, reliability statistics, and linear mixed-effects models accounting for bilateral measurements within participants. No significant differences were observed between the left and right eyes for aFID, vFID, or aCON. Bland–Altman analysis showed no systematic interocular bias across DRVA-derived parameters, although the limits of agreement were widest for aFID, indicating greater interocular variability. Relative reliability was highest for vFID, followed by aCON, whereas aFID showed only fair agreement. Similarly, absolute reliability analyses identified vFID as the most stable biomarker, with the lowest coefficient of variation, while aFID demonstrated the greatest variability. Linear mixed-effects models showed no significant moderating effects of eye dominance, ˙̇O_2_ peak, or IOP on aFID, vFID, or aCON. These findings suggest that retinal vascular endothelial responses measured by DRVA are not systematically influenced by eye dominance or selected systemic physiological factors in healthy young adults. However, given the observed interocular variability, particularly for aFID, assessment of both eyes should be considered in clinical and research settings to improve measurement precision and reproducibility.

## Introduction

Dynamic retinal vessel analyzer (DRVA) is a novel, non-invasive imaging technique that evaluates vascular endothelial responses by measuring real-time changes in retinal vessel diameter during flicker-light provocation [1]. It provides a unique insight into cardiometabolic health, enabling the assessment of the dilation and constriction capacities of the retinal vasculature and the early detection of pathohistological remodeling of the microvasculature [1–3]. Recent literature has indicated that a reduction in flicker-light dilation (FID) correlates with increased cardiometabolic risks, including hypertension, type 2 diabetes, kidney diseases, and obesity [4–6]. Conversely, recent studies in clinical populations have demonstrated that aerobic exercise significantly enhances microvascular function as measured by DRVA [7–9]. For instance, our recent research revealed that an 8-week high-intensity interval training (HIIT) intervention led to an 11% improvement in peak oxygen uptake (˙̇O_2_ peak) in a patient with choroidal melanoma, accompanied by a two-fold increase in both arteriolar and venular dilation in response to FID stimulation [9]. Furthermore, the retinal arteriolar flicker-induced dilation (aFID) has been identified as a sensitive biomarker for assessing short-term exercise effectiveness; Twerenbold et al. [8] reported enhancements in microvascular endothelial function following 8 weeks of HIIT in older hypertensive patients. According to the authors, these improvements surpassed those observed in large conduit arteries assessed via the gold-standard flow-mediated dilation (FMD) technique. Additionally, the same research group demonstrated good to excellent day-to-day and interobserver reliability (e.g., the intraclass correlation coefficient [ICC]) of maximum aFID and venular flicker-light induced dilation (vFID) in a sample of twenty-six middle-aged men and women [10]. Notably, data on absolute reliability measures, such as the coefficient of variation (CV%) and standard error of measurement (SEM), have yet to be presented in the literature, leaving a gap in understanding the measurement resolution (i.e., consistency and reliability) of the device. While the DRVA technique is a validated surrogate biomarker for systemic health, challenges remain. For example, there is a lack of standardized eye selection protocols; current research often relies on the arbitrary selection of a single eye, typically the right, without a standardized physiological justification [3–8]. Although reference values for FID of the right eye exist [3], there is a critical gap in comprehensive data analysis regarding the moderating effects of systemic factors such as ˙̇O_2_ peak, intraocular pressure (IOP), and biological eye dominance. Therefore, one aim of this study is to examine (i) interocular symmetry and its reliability in both absolute and relative terms, and (ii) the moderating effects of systemic factors like ˙̇O_2_ peak, IOP, and biological eye dominance on local vascular beds and their responses to FID stimuli. Our research seeks to determine whether the current practice of arbitrary eye selection is scientifically justified or if specific physiological moderators must be considered to ensure the accuracy and reproducibility of DRVA measurements. We hypothesize that retinal vascular reactivity is not perfectly symmetrical; specifically, the dominant eye will exhibit a distinct microvascular response profile compared to the non-dominant eye. This suggests that eye selection should be standardized based on dominance rather than convenience. We further believe that individual physiological characteristics, such as aerobic fitness (˙̇O_2_ peak) and resting IOP, will moderate the magnitude of the flicker-induced response. Consequently, FID results cannot be accurately interpreted without accounting for these systemic variables, and sensitivity to these moderators will likely differ among vessel types, with vFID and arterial constriction (aCON) exhibiting different degrees of variation compared to aFID.

## Materials and methods

This study is a prospective methodological investigation examining the measurement resolution of the DRVA device and the potential effects of moderators such as ˙̇O_2_ peak, IOP, and eye dominance. Data were collected during two separate laboratory visits. Following medical clearance, participants were advised to refrain from excessive physical activity, caffeine, or alcohol for at least 24 h prior to data collection. During the first visit, all participants performed a cardiopulmonary exercise test (CPET) to voluntary exhaustion, while in the second visit, their IOP, resting blood pressure, and vascular endothelial function were assessed using the DRVA for both the left and right eyes in random order.

### Study participants

Participants were recruited through word of mouth, social media advertisements, and flyers distributed throughout the University Hospital and Faculty of Medicine. Inclusion criteria necessitated that participants be apparently healthy men and women aged 18 years and older, with no history of eye disease, cardiovascular conditions, metabolic disorders, or any chronic diseases, including obesity (defined as a body mass index [BMI] greater than 34.9 kg/m^2^). Exclusion criteria included diabetic retinopathy, peripheral arterial disease, any known neuromuscular injury, current smoking, and the use of oral contraceptives, medications, or dietary supplements (e.g., creatine, whey protein, or nitric oxide boosters). Additionally, individuals with acute orthopedic issues or contraindications to exercise were excluded [11]. All participants meeting the inclusion criteria provided medical clearance and written informed consent prior to data collection.

### Cardiopulmonary exercise testing

Data were collected at the University Hospital Cologne, in the diagnostics laboratory of the Exercise Oncology Department. The aerobic capacity of participants was assessed using an Ergoline 900 cycle ergometer (Hamburg, Germany) connected to a metabolic chart (MetaLyzer 3B system, Cortex, Leipzig, Germany) in accordance with previously published protocols [9, 11, 12]. After collecting resting vital signs, including heart rate, blood pressure, and peripheral capillary oxygen saturation (SpO_2_), participants were fitted with a silicone mask (Hans Rudolph, Kansas City, USA) that was connected to a metabolic chart and a Polar H10 heart rate monitor (Polar Oy, Finland). A standardized ramp protocol was implemented, beginning with a 3-minute rest period followed by an initial workload of 30 W, which increased by 15 W per minute until volitional exhaustion was reached. Task failure was defined as the inability to maintain the required cadence of 70 rpm for more than 10 s. Breath-by-breath gas exchange was monitored throughout the test using a calibrated metabolic chart (MetaLyzer 3B system) to determine systemic gas exchange, peak power output (PPO), and maximal heart rate (HR max).

**Figure 1.**
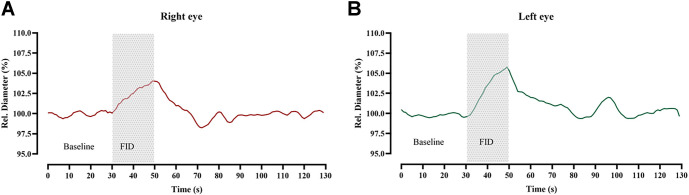
**Representative retinal vessel diameter response during DRVA assessment.** Representative time-course traces of relative retinal vessel diameter recorded during DRVA assessment in the right eye (A) and left eye (B) of a single participant. The shaded area indicates the flicker-light stimulation period used to provoke retinal vessel dilation, following the baseline recording phase. Data are expressed as relative vessel diameter change (%) and were averaged over 5-second intervals. Abbreviations: DRVA, dynamic retinal vessel analyzer; FID, flicker-induced dilation.

### DRVA

Assessment of vascular endothelial function in the retina for both the left and right eyes was performed using the DRVA device (Imedos Health GmbH, Jena, Germany). This technique is widely recognized for inducing retinal vessel dilation through the stimulation of an optoelectronic shutter within the retinal camera [1–3]. Prior to DRVA assessment, we measured best-corrected distance visual acuity using an automatic refractometer (ARK-1s, Nidek, Tokyo, Japan) and IOP using rebound tonometry (ic100, Icare, Vanda, Finland). To determine eye dominance, we applied a simple ring-bearing test: the participant looks at the examiner’s nose from a distance of three meters through a circle created by the subject’s overlapping hands with arms outstretched. The eye visible through the circle is identified as the dominant eye [13]. The DRVA data collection protocol began with resting blood pressure measurement and the application of tropicamide (1%) to provoke pupil dilation. Participants were then instructed to focus on a green cross positioned within the camera and to remain still during the measurement. The measurement process began with a baseline recording lasting 50 s, followed by 350 s during which three phases of flicker-light lasting 20 s each were applied to provoke vessel dilation by overstimulating retinal photoreceptors at a frequency of 25 Hz. Changes in retinal diameter resulting from the applied stimulus were automatically computed using the integrated Retinal Vessel Analyzer software (RVA 4.61; IMEDOS Systems GmbH, Jena, Germany) as a function of diameter change relative to the individual baseline. To quantify maximum dilation, we applied the protocol recently published by Streese et al. [3]. One venule and one artery were selected from each eye’s superior or inferior vascular arch based on measurability criteria (e.g., branch density, clearance to adjacent vessels). Subsequently, the following variables were derived from the device: aFID, vFID and aCON in accordance with the manufacturer’s guidelines. A representative trace of the DRVA measurement is presented in Figure 1 for both eyes.

### Ethical statement

The Institutional Review Board of the Medical Faculty at the University of Cologne approved this study (No. 13-050), conducted within the framework of the Oncological Exercise Therapy (OTT) initiative established at University Hospital Cologne [14]. All procedures adhered to the Declaration of Helsinki (version from 2013) and Good Clinical Practice guidelines.

### Statistical analysis

All data were analyzed using the JAMOVI open-access tool (version 2.7.6., www.jamovi.org) and are presented as mean ± standard deviation (SD), while graphs were created using GraphPad Prism version 11.00 (Graph-Pad Software, La Jolla, CA, USA). The normality of the data distribution was assessed using the Shapiro–Wilk test. A paired Student’s t-test was employed to evaluate interocular symmetry. The relative reliability of all dependent DRVA-derived variables was calculated using the ICC, two-way random model with 95% confidence intervals (CIs). Reproducibility was categorized as poor with an ICC ≤ 0.20, fair between 0.21 and 0.40, satisfactory between 0.41 and 0.61, good between 0.61 and 0.80, and excellent with an ICC ≥ 0.81 [12]. The SEM and CV (%) were provided as measures of absolute reliability. To detect any potential systemic bias, Bland–Altman plots were generated. To evaluate the influence of potential moderators on DRVA-derived parameters, linear mixed-effects models (LMM) were performed using the GAMLj module. This approach was selected to account for the two eyes per participant, thereby controlling for interocular dependency while maximizing statistical power within a limited sample size (*n* ═ 20). Separate models were constructed for each dependent DRVA variable: aFID, vFID, and aCON. In each model, ˙̇O_2_ peak and IOP were entered as fixed-effect covariates to determine their respective contributions to the variance in vessel response. To account for the correlated nature of the bilateral measurements, a participant ID was included as a random effect (random intercept). Parameters were estimated using the restricted maximum likelihood (REML) method. The significance of fixed effects was assessed using Satterthwaite’s approximation for degrees of freedom. Normality and homoscedasticity of the residuals were verified through visual inspection of quantile–quantile plot (Q-Q plot) and residual-versus-predicted value plots. Statistical significance was set at *P* < 0.05, using a two-tailed approach.

**Table 1:** Biometric characteristics of study participants

	***n* ═ 20 (55%)**	**95% CI**
Age, years	30 ± 5	28--32
Body height, cm	178 ± 9	174--182
Body mass, kg	75 ± 12	69--79
BMI, kg·m^-2^	23.4 ± 2.4	22.3--24.4
Resting MAP, mmHg	84 ± 8	80--87
Resting IOP, L eye, mmHg	13.9 ± 3.6	12.3--15.4
Resting IOP, R eye, mmHg	13.5 ± 3.8	11.8--15.1
˙̇O_2_ peak (mL·min^-1^·kg^-1^)	39.6 ± 6.3	36.8--42.4
Peak power output, W	245 ± 55	221--270
HR max., bpm	175 ± 8	171--178

Data are presented as mean ± SD. Abbreviations: BMI, body mass index; bpm, beats per minute; CI, confidence interval: HR max;, maximum heart rate; IOP, intraocular pressure; L, left; MAP, mean arterial pressure; R, right; ˙̇O_2_ peak, peak oxygen uptake.

**Table 2:** Reliability analysis of parameters from the dynamic retina vessel analyzer

	**Right eye**	**Left eye**	**T-test**	***P* value**	***Cohens d***	**ICC**	**95% CI**	**SEM**	**CV (%)**
*aFID*	4.11 ± 1.82	3.92 ± 2.31	0.288	0.775	0.09	0.377	(--0.354--0.709)	1.66	46.4
*vFID^#^*	5.27 ± 2.13	5.00 ± 1.99	0.414	0.681	0.13	0.662	(0.276--0.843)	1.19	28.9
*aCON*	--2.62 ± 1.37	--2.38 ± 1.22	0.582	0.564	--0.18	0.504	(--0.064--0.770)	0.49	42.8

The symbol # indicates that data for the right eye is missing for *n*= 19; for all other variables, *n* ═ 20. Abbreviations: aCON, arteriolar constriction; aFID, arteriolar flicker-induced dilation; CI, confidence interval; CV, coefficient of variation; ICC, intraclass correlation coefficient; SEM, standard error of measurement; vFID, venular flicker-induced dilation.

## Results

**Figure 2.**
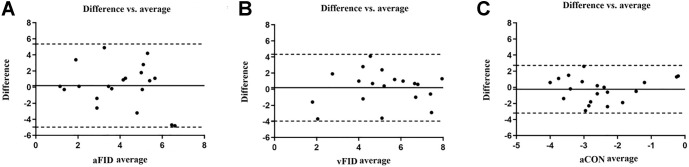
**Bland–Altman analysis of interocular agreement for DRVA-derived biomarkers.** Bland–Altman plots showing interocular agreement for arteriolar flicker-induced dilation (A), venular flicker-induced dilation (B), and arteriolar constriction (C). The x-axis represents the mean value of both eyes for each parameter, while the y-axis represents the interocular difference between eyes. Each dot represents one participant-level comparison. The solid horizontal line indicates the mean interocular difference, reflecting systematic bias, whereas the dashed lines indicate the upper and lower 95% limits of agreement. Abbreviations: aCON, arteriolar constriction; aFID, arteriolar flicker-induced dilation; DRVA, dynamic retinal vessel analyzer; vFID, venular flicker-induced dilation.

**Figure 3.**
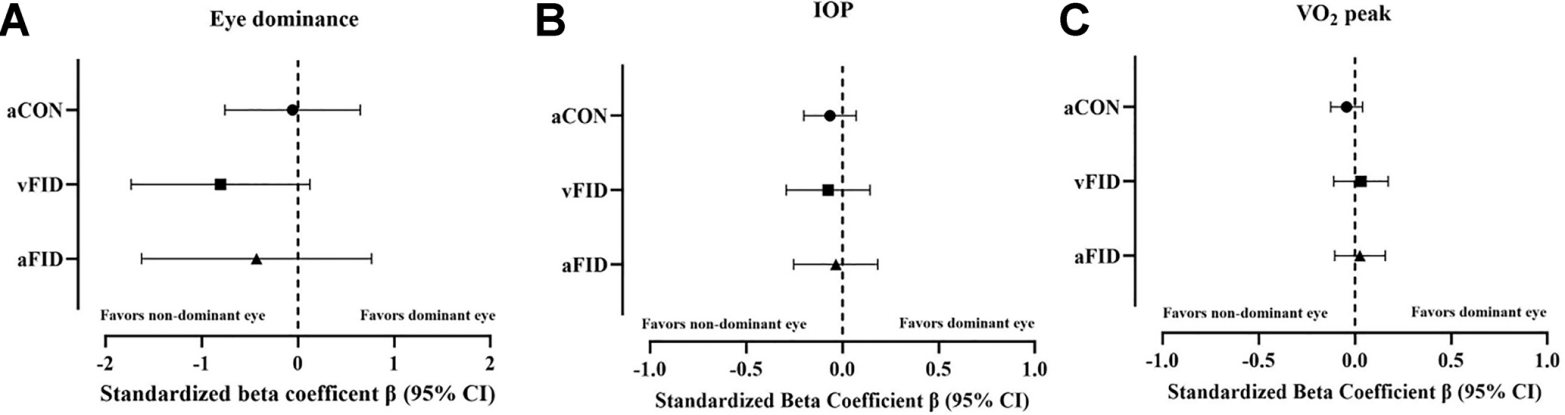
**Linear mixed-effects model analysis of biological and systemic moderators of DRVA-derived biomarkers.** Forest plots showing the standardized beta coefficients and 95% confidence intervals for the association of eye dominance (A), intraocular pressure (B), and peak oxygen uptake (C) with arteriolar flicker-induced dilation, venular flicker-induced dilation, and arteriolar constriction. The vertical dashed line represents the null effect. Estimates crossing the null line indicate no significant moderating effect of the examined variable on DRVA-derived retinal vascular responses. Abbreviations: aCON, arteriolar constriction; aFID, arteriolar flicker-induced dilation; CI, confidence interval; DRVA, dynamic retinal vessel analyzer; IOP, intraocular pressure; vFID, venular flicker-induced dilation; ˙̇O_2_ peak, peak oxygen uptake.

The study population consisted of 20 healthy young men and women, with baseline biometric characteristics summarized in Table 1. Initially, 25 participants met the inclusion criteria and provided written informed consent from a baseline pool of 28 individuals. Of the five participants excluded from the final analysis, two were removed due to elevated brightness sensitivity (e.g., the flicker-light stimulus), while three withdrew from the study without providing a reason. The overall quality of the FID signal, as measured by the internal metric provided by the manufacturer, exceeded 70% for both the veins and arteries of both eyes. Data on the vFID response for one participant were lost due to a technical error affecting the right eye. Participants included in the data analysis exhibited normal BMI, resting blood pressure, and IOP, while their ˙̇O_2_ peak was slightly higher compared to age-matched normative values. *t*-test analysis revealed no significant differences in aFID (*P* ═ 0.775), vFID (*P ═* 0.681), and aCON (*P ═* 0.564) between the left and right eyes. The relative reliability analysis demonstrated good to satisfactory agreement (ICCs for vFID: 0.662 and aCON: 0.504), while aFID exhibited fair reliability (0.377). A similar pattern was observed for absolute measures of reliability (CV% and SEM), with high inter-individual variability evident. aFID showed the highest fluctuation (CV: 46.4%, SEM: 1.66), followed by aCON (CV: 42.8%, SEM: 0.49), while vFID remained the most stable metric with a CV of 28.9% and an SEM of 1.19 (Table 2). Figure 1 presents a representative trace of the FID response recorded during DRVA for both eyes of a single participant, with data averaged over a 5-second interval. Figure 2 illustrates the Bland–Altman analysis, which indicated no systemic bias across all dependent variables (Panels A - C). Specifically, for the aFID parameter, the Bland-Altman analysis revealed a negligible mean bias of 0.190 (95% CI: --1.04 to 1.42), indicating no systematic difference between the eyes. However, the 95% limits of agreement (LOA) were wide, ranging from --4.97 to 5.35. The vFID data demonstrated superior interocular agreement compared to arterial responses, with a negligible mean bias of 0.184 (95% CI: --0.835 to 1.20) and narrower 95% LOA (--3.96 to 4.33). Lastly, the aCON data exhibited the narrowest absolute LOA (--3.21 to 2.73) among all DRVA parameters, with a non-significant mean bias of -0.240 (95% CI: -0.948 to 0.468). The LMM analysis presented in the Forest plot (Figure 3, Panels A–C) suggested that eye dominance did not significantly bias the FID measures (Panel A: aFID *P ═* 0.474, vFID=0.094, and aCON *P ═* 0.876). Additionally, the Forest plot analysis of systemic moderators indicated that IOP and ˙̇O_2_ peak did not influence FID outcomes (Panel B: aFID *P ═* 0.69, vFID=0.652, and aCON *P ═* 0.31; Panel C: aFID *P ═* 0.751, vFID=0.492, and aCON *P ═* 0.344). Regarding model diagnostics, reliability was highest for the vFID model (Conditional R^2^=0.68, 95% CI: --1.731–0.124), followed by aCON (Conditional R^2^=0.52, 95% CI: --0.758–0.649), while the aFID model (Conditional R^2^=0.38, 95% CI: --1.624–0.766) was more susceptible to individual variation. Visual inspection of Q-Q plots and Shapiro-Wilk tests confirmed that the residuals for all parameters followed a normal distribution, and the absence of distinct patterns in residual vs fitted plots indicated a lack of significant data heterogeneity across all three models.

## Discussion

The aim of this study was to evaluate the interocular symmetry and reliability of retinal vascular reactivity as measured by aFID, vFID and aCON, while also assessing the influence of biological and systemic moderators on these biomarkers. We hypothesized that retinal vascular reactivity would not be perfectly symmetrical, suggesting that the dominant eye would exhibit a distinct microvascular response profile compared to the non-dominant eye, thereby affecting the eye selection procedure. Furthermore, we hypothesized that individual physiological characteristics, such as ˙̇O_2_ peak and resting IOP, would significantly modulate the magnitude of the flicker-induced response, influencing different small blood vessels with varying degrees of response. The main findings of this study align partially with our initial hypotheses, as we observed satisfactory to good relative reliability for aCON and vFID, while aFID showed fair agreement in interocular readings. Importantly, the CIs for all three DRVA-derived variables were quite wide, and given the relatively small sample size, the ICC values obtained should be interpreted with caution. This is also reflected in absolute measures, where vFID remained the most stable metric compared to the high inter-individual variability and fluctuations observed in aCON and aFID (CV > 42%, Table 2). 

These findings on the relative and absolute reliability analysis provide the first evidence in the literature regarding eye symmetry assessed via the DRVA device. Previous research primarily focused on relative day-to-day reliability or correlation analysis [10]. A recent explanation for the variation among FID biomarkers was proposed by Streese et al. [10], who reported satisfactory to good relative intra-day and intra-observer variability in FID response of the right eye. They suggested that percentage-based methods, such as aFID, should be corrected for baseline diameters. This approach is recommended by other experts in the field [15] and has already been integrated into FMD analyses, which also measures vascular endothelial function of large conduit arteries [8]. Nevertheless, the observed variations in retinal FID parameters are pertinent for both preclinical and clinical research, as aFID serves as a sensitive biomarker for tracking microvascular adaptations to exercise interventions [7–9]. In clinical research, early signs of microvascular dysfunction and blood vessel remodeling manifest in retinal vessels long before the clinical onset of various cardiometabolic diseases [1]. This is particularly significant in the field of microvascular circulation and its integration into clinical medicine [16, 17]. However, as this was an observational study in a growing field of research, we cannot provide a straightforward explanation for the sources of biological variation in primary outcome variables. We can outline that the data presented on FID response aligns with reference values provided by Streese et al. [3] for the respective age and fitness level group.

Importantly, the Bland-Altman analysis (Figure 2, panels A-C) revealed a small mean bias for all three dependent variables, indicating no systematic difference between eyes. The mean differences were minimal (Figure 2), and the 95% LOA varied by metric; aCON (--3.21 to 2.73) and vFID (--3.96 to 4.33) demonstrated relatively tight intervals, while aFID exhibited substantially wider limits (--4.97 to 5.35). These results suggest that while eye selection does not introduce directional bias, venous and contrast-based metrics provide superior interocular precision compared to arterial dilation, indicating that measurements between eyes should not be considered interchangeable for clinical decision-making. Previous studies in this area have focused on the arbitrary selection of the right eye [2–5, 7, 8] or the dominant eye as noted by Günthner et al. [6]. To the best of our knowledge, only one original study on cardiovascular patients tested both eyes using the DRVA device [18], aiming to identify early signs of major adverse cardiovascular events. Here, we aimed to comprehensively evaluate interocular symmetry and the influence of systemic factors on DRVA accuracy in healthy individuals. According to the mixed model analysis, the microvascular FID response remained independent of eye dominance and systemic physiological moderators, indicating no clear evidence of these effects in the studied population (Figure 3, panels A-C). This finding may be attributed to our examination of healthy, normotensive, aerobically fit individuals, which revealed no confounding effects on FID. Previous research indicates that changes in microvascular function and aerobic fitness often coincide due to exercise-induced increases in nitric oxide bioavailability. Consequently, researchers frequently report approximately 25%–30% shared variance between measurements of small and large conduit arteries and systemic O_2_ uptake measured via CPET [7, 19]. Thus, it would be valuable to investigate whether such findings are also present in sedentary, aging, or clinical populations, where the influence of confounders is anticipated [20]. This certainly requires further investigation.

Some limitations of our study should be acknowledged. The sample size was relatively small and consisted solely of healthy individuals; therefore, these conclusions should not be generalized to clinical populations. To clarify our approach and justify the limited sample size, we examined both eyes, resulting in a data collection and analysis workload that was double that of similar studies in this field. For instance, the normative DRVA data study by Streese et al. [3] for this age group assessed 39 participants, resulting in 39 measurements and data sets analyzed. In our study, we reported the same amount of workload divided between two eyes to comprehensively evaluate factors influencing DRVA assessments in a healthy population.

## Conclusion

This study is the first to comprehensively evaluate the absolute and relative reliability of DRVA-derived biomarkers, as well as the influence of potential moderators. The vFID biomarker exhibited the highest absolute and relative reliability, followed by aCON, while aFID was identified as the least consistent and symmetrical parameter. Our key conclusion is that there is no clear evidence that the vascular endothelial response of the retina is influenced by biological eye dominance or systemic factors within this population. Data from the relative reliability analysis indicate that CIs for all three DRVA-derived variables were relatively wide (aFID: -0.355–0.709; vFID: 0.276–0.843; aCON: -0.064–0.770). Given the relatively small sample size, the interocular ICC readings observed here should be interpreted with caution. Nevertheless, to enhance diagnostic precision and the detection of cardiovascular risk factors derived from FID assessment, we recommend assessing both eyes in clinical practice. This approach will improve data reliability, especially considering the significant interocular variability observed, particularly for the aFID parameter, which is the most commonly used biomarker in exercise intervention studies. Notably, this study represents an initial step toward future methodological research, and its findings should not be generalized to other populations.

## Acknowledgement

The Dynamic retina vessel analyzer device was provided by the A.M. Wilsing Stiftung to the Department of Cardiology, Heart Center, Faculty of Medicine, University of Cologne, DE.

## Data Availability

The data collected and analyzed in this study are not publicly available due to privacy and ethical constraints. However, they can be obtained from the corresponding author upon a reasonable request.
